# Molecular Phenotyping of Telomerized Human Bone Marrow Skeletal Stem Cells Reveals a Genetic Program of Enhanced Proliferation and Maintenance of Differentiation Responses

**DOI:** 10.1002/jbm4.10050

**Published:** 2018-05-24

**Authors:** Natalie A Twine, Linda Harkness, James Adjaye, Abdullah Aldahmash, Marc R Wilkins, Moustapha Kassem

**Affiliations:** ^1^ Systems Biology Initiative School of Biotechnology and Biomolecular Sciences University of New South Wales Sydney Australia; ^2^ CSIRO Sydney Australia; ^3^ Department of Endocrinology and Metabolism Endocrine Research Laboratory (KMEB) Odense University Hospital Odense Denmark; ^4^ Institute for Stem Cell Research and Regenerative Medicine Faculty of Medicine Heinrich Heine University Düsseldorf Germany; ^5^ Stem Cell Unit Department of Anatomy, Faculty of Medicine King Saud University Riyadh Saudi Arabia

**Keywords:** OSTEOBLASTS, BONE MARROW STROMAL (SKELETAL) STEM CELLS (hMSC), HMSC‐TERT, TELOMERIZATION, MOLECULAR PHENOTYPE

## Abstract

Long‐term in vitro expansion of bone marrow stromal (skeletal) stem cells (also known as human mesenchymal stem cells [hMSC]) is associated with replicative senescence and impaired functions. We have previously reported that telomerization of hMSC through hTERT overexpression led to bypassing a replicative senescence phenotype and improved in vitro and in vivo functions. However, the molecular consequence of telomerization is poorly characterized. Thus, we compared the molecular phenotype of a well‐studied telomerized hMSC (hMSC‐TERT) cell line with primary hMSC. At a cellular level, both cell populations exhibited strong concordance for the known hMSC CD markers, similar responses to osteoblast (OB) differentiation induction, and formed heterotopic bone in vivo. Overall gene expression was highly correlated between both cell types with an average Pearson's correlation coefficient (R^2^) between the gene expression of all primary hMSC and all hMSC‐TERT samples of 0.95 (range 0.93–0.96). Quantitative analysis of gene expression of CD markers, OB cell markers, and transcription factors (TF) showed a high degree of similarity between the two cell populations (72%, 77%, and 81%, respectively). The hMSC‐TERT population was enriched mainly for genes associated with cell cycle and cell cycle signaling when compared with primary hMSC. Other enrichment was observed for genes involved in cell adhesion and skeletal system development and immune response pathways. Interestingly, hMSC‐TERT shared a telomerization signature with upregulation of cancer/testis antigens, MAGE, and PAGE genes. Our data demonstrate that the enhanced biological characteristics of hMSC after telomerization are mainly due to enhanced expression of cell proliferation genes, whereas gene expression responses to differentiation are maintained. © 2018 The Authors. *JBMR Plus* Published by Wiley Periodicals, Inc. on behalf of the American Society for Bone and Mineral Research

## Introduction

Human bone marrow‐derived skeletal (also known as stromal or mesenchymal stem cells; hMSC) are multipotent adult stem cells. They are present in the bone marrow stroma and capable of self‐renewal and multi‐lineage differentiation into mesoderm‐type cells such as osteoblasts, adipocytes, and chondrocytes.^1^, ^2^ In addition, hMSC exhibit immune‐modulatory and regeneration‐enhancing characteristics based on secretion of a large number of molecules.^3^ These qualities have encouraged clinical testing of hMSC for the enhancement of tissue regeneration after injury. As reviewed previously,^4^ hMSC have been examined for their potential use in repair of bone defect and cartilage defects, enhancing tissue regeneration in ischemic heart disease, after acute renal injury as well as for treatment of steroid‐resistant graft versus host disease.

One of the major challenges of cellular therapeutics is how to generate large numbers of normal cells, through enhancing cell proliferation, while maintaining their biological and differentiation responses. Normal diploid cells, including hMSCs, exhibit a replicative senescence phenotype when cultured in vitro with an associated impairment of cellular functions.^5^, ^6^ Because the main mechanisms leading to in vitro replicative senescence is telomere shortening, caused by the absence of telomerase activity,^7^ cellular telomerization has been suggested as an approach to generate cells suitable for therapies, eg, tissue engineering.^8^ We have previously reported the generation and characterization of telomerized hMSC, which we termed hMSC‐TERT, and reported that telomerization abolished replicative senescence and not only maintained but enhanced bone formation capacity of the cells when implanted subcutaneously in immune‐deficient mice.^9^ Other investigators corroborated these findings^10^, ^11^, ^12^, ^13^, ^14^, ^15^ (see summary of the telomerized cell lines in Table 1). Telomerized hMSC are also good cellular models for primary hMSC because of their stable phenotype and are thus suitable for proteomic and genomic studies requiring the use of a large number of cells.^16^, ^17^, ^18^ Conversely, telomerization may change the biology of hMSCs and may affect their relevance as a representative model for primary hMSC. To help understand this, global molecular phenotyping techniques such as DNA microarrays can be used. This method has been applied to the unbiased classification of cancer subtypes, as described in the landmark study by Golub and colleagues,^19^ but it has not been widely used within the hMSC field. Thus, we performed molecular phenotyping using microarrays to compare primary hMSC and telomerized hMSC‐TERT cells that have previously been created in our group^9^ and have been utilized in many studies by us and by others (more than 100 studies to date). We investigated the molecular pathways associated with the enhanced biological functions of telomerized hMSC and sought to clarify the relevance of these cells as cell models for primary hMSC. We investigated molecular phenotype by examining a set of osteoblast (OB) gene markers that are differentiation stage‐specific,^20^ along with genes for cell surface markers, transcription factors, immune molecules, and signaling pathways.

**Table 1:** Summary of Studies Investigating Immortalization of hMSC

Source of MSC	Reference	Method	Extended life span	Differentiation capacity (OB/AD/CC or other lineages)	In vivo implantation	Tumorigenicity in vivo/in vitro
Adipocytes	Balducci et al., 2014	Adipose‐derived stem cells (hASCs) transduced with hTERT and SV‐40/HPV E6/E7	hASCs‐TS and hASCs‐TE cultured for 1 year with a population doubling level up to 100	Immortalized hASC‐TS but not hASC‐TE significantly reduced differentiation potential in OB and AD lineage	n/a	hASC‐TERT did not show tumorigenic properties in vitro
Bone marrow	Skarn et al., 2014	Telomerization of BM‐MSC to form iMSC3# cell line	Maintained in long‐term culture (155 population doublings)	iMSC#3 maintained their capacity to differentiate into OB, AD, and CC lineages	n/a	iMSC#3 had a normal karyotype; no tumor formation after 6 months of implantation of iMSC#3 in mice
Bone marrow	Dai et al., 2017	hMSCs transfected with hTERT and CTLA4Ig	hTERT‐MSC and hTERT‐CTLAIg hMSCs showed extended life span in culture compared with hMSC	hTERT‐CTLAIg hMSCs retained OB differentiation capacity	hTERT‐CTLA4Ig hMSCs formed bone (in vivo) when transplanted in rat	hTERT‐CTA4Ig hMSCs did not form tumors after in vivo transplantation
Bone marrow	Piper et al., 2012	Inducible Tet‐On gene expression system to create immortalized hMSCs (i‐hTERT hMSCs)	i‐hTERT hMSCs able to proliferate significantly longer than primary hMSCs	i‐hTERT hMSCs retain multipotentiality (OB, AD, CC) in vitro	n/a	This cell line allows inducible expression of telomerase, therefore avoiding tumor formation
Bone marrow	Tsai et al., 2010	Ectopic overexpression hMSCs with HPV 16 E6E7 and hTERT	Telomerized cells overcome senescence and could be passaged over 100 population doublings	hMSC‐HPV‐TERT had enhanced differentiation potential (OB and neural lineages); maintained differentation potential in CC and AD lineages	n/a	n/a
Fetal porcine pancreas	Cao et al., 2011	Transfected MSC with hTERT, resulting in immortalized cell line, iPMSCs	The iPMSCs have been cultured for more than 80 passages	Retain capacity for differentiation into neurons, cardiomyocytes, germ cells, and islet‐like cells	Transplantation of iPMSCs to diabetic mice may have the potential to mimic the normal physiological insulin response	iPMSCs were transplanted into mice; no tumors were observed for up to 2 months after injection
Amniotic cells/adipocytes	Wolbank et al., 2009	Immortalized by ectopic expression of hTERT	Telomerized MSC expanded to at least PD60 with no signs of growth retardation	Similar or enhanced differentiation potential to OB and AD lineage compared with primary cells	n/a	Telomerized MSCs showed a normal karyotype
Bone marrow	Huang et al., 2008	hMSCs transduced with exogenous hTERT (hTERT‐hMSCs	hTERT‐MSC cultured for 290 population doublings	hTERT‐MSCs maintained ability to differentiate into AD, CC, and OB	n/a	hTERT‐MSCs were non‐tumorigenic in vivo; karyotype analysis of hTERT‐MSCs were normal
Bone marrow	Liu et al., 2013	MSCs immortalized using p53 and hTERT	P53‐hTERT‐MSC maintained primary MSC morphology after 1 year of continuous culture	P53‐hTERT‐MSC retained differentiation potential for AD, CC, and OB	n/a	In vivo transplantation of immortalized MSCs showed no tumor at 12 weeks

## Materials and Methods

### Cell culture

hMSC‐TERT cells were created as described previously, with hMSC obtained from a healthy 30‐year‐old male donor.^9^ The cells exhibit all typical characteristics of hMSC.^9^ The passage number for the hMSC‐TERT cells was p38 and population doubling level (PDL) 80. At this PDL, the cells exhibited a “stemness” phenotype in an in vivo heterotopic bone formation assay.^21^ hMSC‐TERT cells were cultured in minimal essential media (MEM, Invitrogen, Carlsbad, CA, USA) with 10% v/v fetal bovine serum (FBS, PAA, Pasching, Austria).

Primary hMSC were derived from bone marrow aspirates taken from the iliac crest of healthy donors between the ages of 20 and 30 years. Both oral and written consent was obtained from each participant, and the project was approved by the regional Scientific Ethical Committee. Isolation of bone marrow mononuclear cells was performed in heparinized MEM using low‐density gradient centrifugation and Lymphoprep (Medinor, Bronby, Denmark).^22^ hMSC were obtained through plastic adherence and cultured and expanded in MEM supplemented with 10% v/v FBS (PAA) and penicillin/streptomycin (Invitrogen) at a seeding density of 12.5 × 10^3^ cells per cm^2^.

### Flow cytometry analysis

Flow cytometry (FACS) analysis of cell surface markers on hMSC‐TERT and primary hMSC was performed as described in Harkness and colleagues.^21^ The preconjugated markers examined were CD44‐PE, CD63‐FITC, CD73‐PE, CD90‐PE, CD105‐PE, CD146‐PE, and CD166‐PE (all BD Pharmingen, San Diego, CA, USA). In brief, subconfluent cells were single‐cell suspended in trypsin, washed twice in FACS buffer (PBS^2‐^, 0.5% BSA), before incubation for 30 minutes on ice with one of the antibodies. After two wash steps with FACS buffer, flow cytometry was performed using a Cell Lab Quanta SC‐MPL and analysis performed using Kaluza 1.1 (both Beckman Coulter, Brea, CA, USA).

### Osteoblastic differentiation

hBSMC‐TERT and primary hMSC were plated at 20,000 cells per cm^2^ in osteoblastic induction medium (OIM) as previously reported.^21^ In brief, MEM was supplemented with 10 mM β‐glycerolphosphate (Calbiochem, EMD Millipore, Billerica, MA, USA), 50 µg/ml L‐ascorbic acid‐2‐phosphate (Wako Chemicals, Richmond, VA, USA), 10 nM dexamethasone (Sigma‐Aldrich, St. Louis, MO, USA), and 10 nM calcitrol (LEO Pharma, Madison, NJ, USA) for a maximum of 15 days. Medium was replaced every 3 days.

Alkaline phosphatase (ALP) activity was measured at day 6 of OIM using Cell Titre Blue (Promega, Madison, WI, USA) to measure cell viability and p‐nitro phenyl phosphate (pNP) to measure ALP activity as previously described.^21^ Absorbance was measured on a FLUOstar Omega plate reader (BMG LabTech, Cary, NC, USA) and ALP activity corrected for cell viability (*n* = 6).

### Immune staining and morphological analysis

hBSMC‐TERT and primary hMSC cultured in OIM were stained for ALP and matrix mineralization (Alizarin red [AZR]) at day 15 of induction as previously described.^23^ In addition, non‐induced cells (control) and cells undergoing OIM were trypsinized (at day 9) and seeded in 96‐well flat‐bottom NUNC dishes at a cell count of 5000/well. The following day, cells were fixed in 10% formalin before staining. Wells were blocked with 5% FBS in phosphate‐buffered saline (PBS^2+^) for 1 hour at room temperature (RT) before overnight incubation with the antibody (ALP [R&D Systems, Minneapolis, MN, USA, or Larry Fischer); Runt related transcription factor [RUNX2, Abcam, Cambridge, MA, USA]). Wells were washed and incubated with an appropriate fluorescent secondary (Alexa Fluor, Life Technologies, Carlsbad, CA, USA) for 1 hour at RT, before counterstaining with 1 μg/mL 4',6‐diamidino‐2‐phenylindole (DAPI) for 15 to 30 minutes at RT. Cells were imaged using an Operatta High Content Screening system (PerkinElmer, Waltham, MA, USA).

### In vivo heterotopic bone formation

hMSC‐TERT cells and primary hMSC (0.5 × 10^6^) were single‐cell suspended and combined with 40 mg hydroxyl‐apatite tricalcium phosphate as previously described.^24^ The cells were incubated overnight in hydroxyapatite‐tricalcium phosphate (HA/TCP) before implantation into the dorsolateral area of immune‐compromised mice (NOD.CB17‐Prkdcscid/J) for 8 weeks. After retrieval, implants were fixed overnight in 4% formalin and washed in PBS before decalcification in formic acid for 3 to 5 days. After embedding in paraffin, sections were cut and stained with hematoxylin and eosin or human specific‐vimentin antibody (#RM‐9120, Clone SP20; Thermo Fisher Scientific, Waltham, MA, USA).

### RNA extraction

Total RNA was isolated using TRIzol (Invitrogen) as previously reported.^9^ After initial extraction, samples were additionally eluted using a GenElute mammalian total RNA miniprep kit (Sigma‐Aldrich; according to manufacturer's instructions) to achieve high‐purity RNA.

### Microarray data generation and analysis

Total RNA was isolated using the GeneMatrix Universal RNA Purification Kit (Cat. E 3598‐02, Roboklon, Berlin, Germany) and quality‐checked by Nanodrop analysis (Nanodrop Technologies, Wilmington, DE, USA). Four hundred nanograms of total RNA was used as input for generating biotin‐labeled cRNA (Ambion, Austin, TX, USA). cRNA samples were then hybridized onto Illumina human‐8 Bead‐Chips version 3. Hybridizations, washing, Cy3‐streptavidin staining, and scanning were performed on the Illumina BeadStation 500 platform (Illumina, San Diego, CA, USA), according to the manufacturer's instruction. hMSC‐TERT RNA samples were analyzed in biological triplicate, and there were 15 samples of primary hMSCs from different individuals. Expression data analysis was carried out using the BeadStudio software 3.0 (Illumina). Raw data were background‐subtracted and normalized using the rank invariant algorithm. Data were then imported into R (version 3.03)^25^ and the BioConductor package “limma”^26^ was used to test for differential expression. Probes that were considered as expressed (detection *p* < 0.01) in at least 3 of the samples were retained for further analysis. Lists of chondrocyte and adipocyte gene markers used in expression analysis were obtained from R&D Systems (https://www.rndsystems.com/research-area/adipogenesis-markers; https://www.rndsystems.com/research-area/chondrogenesis-markers).

### Functional enrichment analysis

The Database for Annotation, Visualization, and Integrated Discovery (DAVID) v6.7^27^ was employed to determine the functional enrichment for sets of differentially expressed genes between hMSC‐TERT and primary hMSC. The filtered set of probes (20,929) identified as expressed in at least 3 samples was used as the background list. MetaCore (v6.20, from Thompson Reuters) was also used for functional enrichment analysis of differentially expressed genes between hMSC‐TERT and primary hMSC. A number of fold‐change thresholds were used to create four lists of differentially regulated genes between hMSC‐TERT and primary hMSC. Thresholds were set at log_2_ fold change of 0.5, 1, 1.5, and 2. All gene lists (summarized in Supplemental Table S1) were generated using an adjusted *p* value threshold of 0.05. Pathways were ranked from most to least significantly enriched for each gene list. The rank for pathways in common across the four gene lists were then summed to indicate which pathways are highly ranked for all gene lists.

## Results

### hMSC‐TERT and primary hMSC exhibit a similar pattern of CD markers and form heterotopic bone in vivo

**Figure 1:**
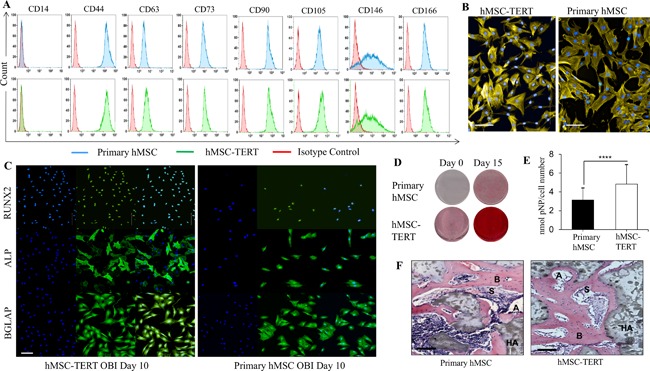
Telomerized human bone marrow skeletal (stromal) stem cells (hMSC‐TERT) exhibit similar phenotype to primary hMSC. (*A*) FACS analysis of canonical CD surface markers expressed in primary hMSC versus hMSC‐TERT. Primary hMSC are indicated in blue, hMSC‐TERT in green, and isotype control in red. (*B*) Primary hMSC and hMSC‐TERT cells exhibit similar morphologies. Cells were stained with F‐actin (Phalloidin‐555) and hoechst. Scale bars = 100 μm. (*C*) Cellular staining for primary hMSC and hMSC‐TERT at day 10 post‐OB induction. For each cell type: nuclear stained DAPI (left); antibody to OB marker RUNX2, ALP, or BGLAP with a secondary FITC antibody (middle); overlayed images (right). Scale bars = 100 μm. (*D*) Alizarin red staining of mineralized matrix formation in primary hMSC and hMSC‐TERT at day 0 and day 15 post‐OB induction. (*E*) Alkaline phosphatase activity at day 6 post‐OB induction of primary hMSC and hMSC‐TERT cells. Data plotted as mean ± SD; *n* = 6 replicates (****p < 0.001). (*F*) In vivo ectopic bone formation after subcutaneously implanted primary hMSC and hMSC‐TERT mixed with hydroxyapatite/tricalcium phosphate (HA/TCP) in immune‐deficient mice. Histological sections of implants with cells after 8 weeks of the implantation stained with H&E. A = adipocyte; S = sinusoid; B = bone; HA= hydroxyapatite. Scale bars = 100 μm.

The cellular phenotype of hMSC‐TERT and primary hMSC was compared using FACS analysis of characteristic hMSC surface markers. As shown in Fig. 1 *A*, there was a similar percentage of positive cells for CD44, CD63, CD73, CD90, CD105, CD146, and CD166, between primary hMSC and hMSC‐TERT cells. Both cell populations were negative for CD14, which is a known marker for monocytes and macrophages.^28^ Primary hMSC and hMSC‐TERT showed similar morphologies (Fig. 1 *B*). In addition, both differentiated readily to OB in vitro as evidenced by expression of OB markers ALP, BGLAP, and RUNX2, formation of mineralized matrix, and increased ALP activity (Fig. 1 *C–E*). hMSC‐TERT and primary hMSC formed heterotopic bone and bone marrow organ when implanted subcutaneously in immune‐deficient mice, corroborating their “stemness” (Fig. 1 *F*).

### hMSC‐TERT and primary hMSC show a similar global gene expression phenotype

**Figure 2:**
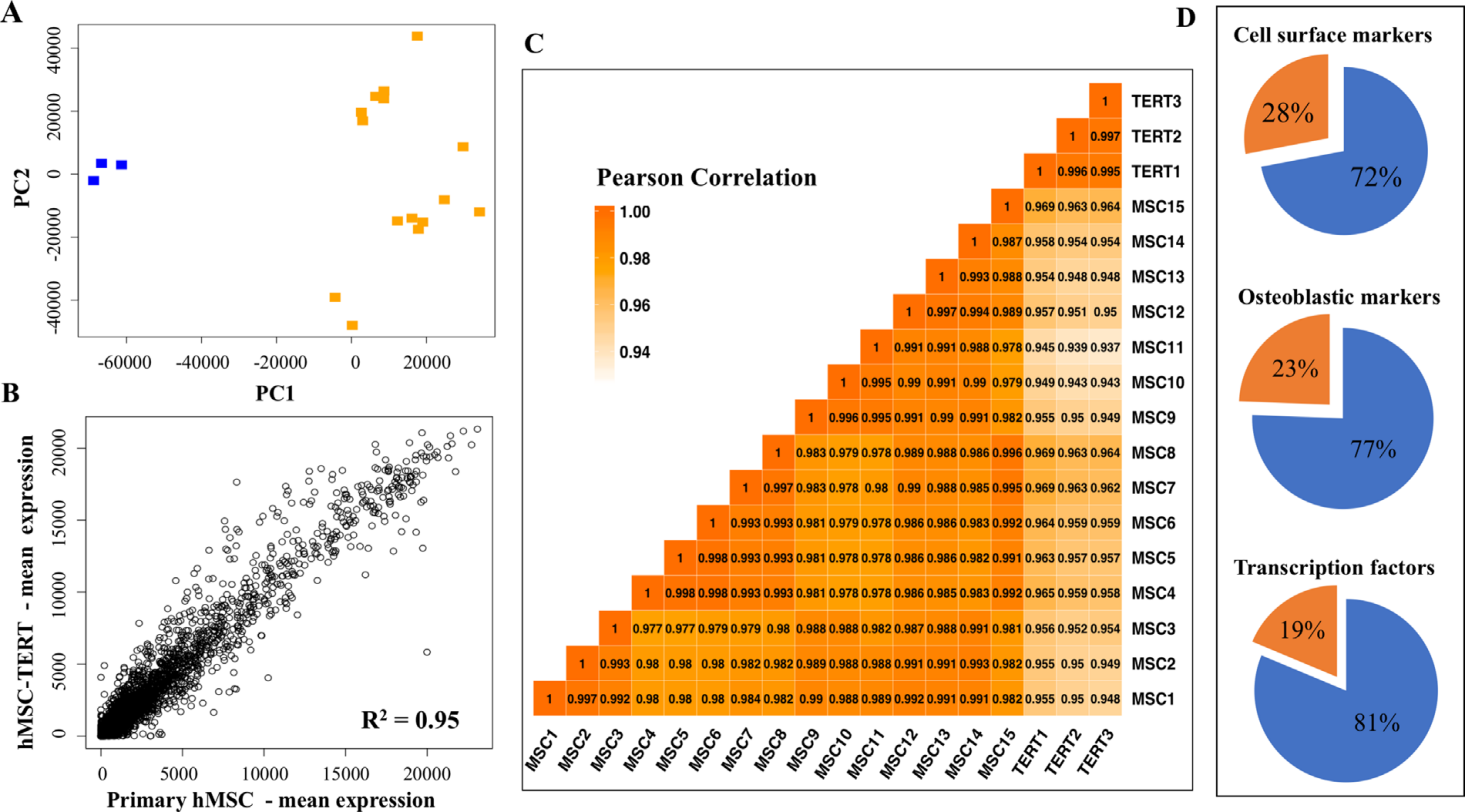
Telomerized human bone marrow skeletal (stromal) stem cells (hMSC‐TERT) exhibit a similar molecular phenotype to primary hMSC. (*A*) Principal components analysis demonstrates that primary hMSC (*n* = 15, orange) samples cluster separately from hMSC‐TERT samples (*n* = 3, blue). (*B*) Scatterplot showing correlation and coefficient of determination (R^2^ value) between global gene expression for all primary hMSC (*n* = 15) and all hMSC‐TERT (*n* = 3) samples. (*C*) Correlation heatmap for all primary hMSC and all hMSC‐TERT samples. The Pearson correlation between each sample is shown and colored according to score. (*D*) Proportional representation for three types of genes differentially regulated between primary hMSC and hMSC‐TERT. Orange proportion refers to genes that are significantly different in gene expression between the cell types, whereas blue refers to genes that are similar in gene expression between the cell types.

We measured the overall similarity in gene expression between all primary hMSC and all hMSC‐TERT samples by correlating their global gene expression profiles from microarrays and performing principal components analysis (PCA). PCA shows the two cell types form discrete clusters, with more variability within primary hMSC than hMSC‐TERT cells (Fig. 2 *A*). For all expressed genes, the average Pearson's correlation coefficient (R^2^) was 0.95 (range 0.93–0.96), while the average R^2^ within primary hMSC samples was 0.98 (range 0.97–0.99) and the average R^2^ value within hMSC‐TERT samples was 0.99 (range 0.99–0.99) (Fig. 2 *B*, *C*). This indicates a very high similarity of global gene expression between the two cell types.

### hMSC‐TERT and primary hMSC exhibit a similar CD marker gene expression phenotype

We next focused on specific groups of genes that are relevant to hMSC biology. We first determined the similarity of expression for a set of CD surface markers, in hMSC‐TERT and primary hMSC. For this, we examined a set of 50 CD markers that were curated from a previous publication; these constitute the core CD marker signature of MSC.^29^ All of the 50 CD markers were expressed in both cell types, consistent with our observations from FACS analysis of a smaller set of markers. There was also strong correlation of gene expression between the hMSC‐TERT and primary hMSC cells (all markers and fold changes are listed in Supplemental Table S2), where a total of 36/50 CD markers (72%) did not show any significant quantitative change in gene expression between hMSC‐TERT cells and primary hMSC (Fig. 2 *D*, Supplemental Table S2). Among the CD markers, 14/50 (28%) were differentially regulated in the hMSC‐TERT compared with primary hMSC (>2 fold change [FC], *p* < 0.05) (Fig. 2 *D*, Table 2). The most dramatically downregulated cell surface markers were ICAM2 (−43.65 FC) and HLA‐DRA (−21.13 FC). Two other CD markers, THY1 (CD90) and CD44, showed significant differences in gene expression (details in Table 2), but this result was not corroborated in our FACS analysis (Fig. 1 *A*). Functional enrichment of the 14 CD markers that show differential gene expression identified wound healing (*p* = 4.24 × 10^−4^) (CD44, CD9, COL3A1, ITGB3) and immune response (*p* = 1.7 × 10^−3^) (CD164, CD97, IL1R1, HLA‐DRA, TNFRSF1B) as functional processes associated with these markers.

**Table 2:** CD and OB Markers Significantly Differentially Regulated Between hMSC‐TERT and Primary hMSC

Gene symbol	Gene name	Fold change	Adjusted *p* value
CD markers			
CD9	CD9	7.13	3.67E‐03
THY1	Thy‐1 cell surface antigen	3.65	1.71E‐03
CD44	CD44	2.56	9.20E‐03
CD97	CD97	2.35	7.57E‐03
CD82	CD82	2.18	4.18E‐04
TNFRSF1B	Tumor necrosis factor receptor superfamily member 1B	−2.13	8.03E‐04
COL3A1	Collagen type III	−2.48	2.10E‐02
CD164	CD164	−2.49	9.98E‐07
IL1R1	Interleukin 1 receptor type 1	−2.92	4.88E‐02
PVRL2	Nectin cell adhesion molecule 2	−3.62	2.35E‐02
CD109	CD109	−7.36	6.47E‐07
ITGB3	Integrin subunit beta 3	−9.03	6.89E‐06
HLA‐DRA	Major histocompatibility complex, class II, DR alpha	−21.13	5.45E‐04
ICAM2	Intercellular adhesion molecule 2	–43.65	3.36E‐04
OB markers			
COL4A5	Collagen type IV alpha 5	317.56	4.78E‐06
CD24	CD24	57.30	2.91E‐06
BMP4	Bone morphogenic protein 4	9.21	1.43E‐03
ALPL	Alkaline phosphatase	7.58	3.57E‐02
CLEC3B	C‐type lectin domain family 3 member B	7.37	2.24E‐03
MFAP5	Microfibrillar associated protein 5	3.82	2.96E‐02
SCARB1	Scavenger receptor class b member 1	3.65	1.22E‐05
OAS3	2'‐5'‐oligoadenylate synthetase 3	3.16	4.76E‐02
COL7A1	Collagen type VII alpha 1	3.04	1.30E‐05
IFIT1	Interferon induced protein with tetratricopeptide repeats 1	2.89	1.46E‐02
HSPG2	Heparan sulfate proteoglycan 2	2.68	2.16E‐02
ITGA7	Integrin subunit alpha 7	2.52	4.71E‐04
OAS1	2'‐5'‐oligoadenylate synthetase 1	2.38	1.08E‐03
EPHA2	EPH receptor A2	2.22	8.47E‐03
FBN2	Fibrillin 2	2.22	2.06E‐02
COL3A1	Collagen type III alpha 1	−2.48	2.10E‐02
CTHRC1	Collagen triple helix repeat containing 1	−2.52	6.55E‐04
WWOX	WW domain containing oxidoreductase	−2.55	1.26E‐02
PDGFA	Platelet derived growth factor subunit A	−2.63	1.36E‐02
ICAM1	Intercellular adhesion molecule 1	−2.88	1.41E‐05
COL8A2	Collagen type VIII alpha 2	−3.09	3.56E‐02
PLOD2	Procollagen‐lysine, 2‐oxoglutarate 5‐dioxygenase 2	−3.32	6.80E‐06
IGF2	Insulin like growth factor 2	−3.72	2.91E‐02
POSTN	Periostin	−3.79	6.95E‐05
TGFB2	Transforming growth factor beta 2	−5.01	1.55E‐02
SGCD	Sarcoglycan delta	−6.17	1.33E‐02
BST2	Bone marrow stromal cell antigen 2	−6.37	8.69E‐07
COL18A1	Collagen type XVIII alpha 1	−8.80	5.64E‐04

### hMSC‐TERT and primary hMSC share a similar pattern of osteoblastic gene expression phenotype

The hMSC‐TERT and primary hMSC were next compared for expression of a set of 123 recently defined OB markers that reflect the differentiating osteoblastic phenotype.^20^ All of the 123 OB markers were expressed in both hMSC‐TERT and primary hMSC and 95/123 (77%) of the markers showed no significant difference in expression between the two cell populations (Fig. 2 *D*). The remaining 28 (23%) OB markers were differentially regulated between primary hMSC and hMSC‐ TERT (>2 FC or <−2 FC, *p *< 0.05). These 28 OB markers, their gene symbols, fold changes and *p* values are detailed in Table 2. All OB markers and associated fold change and *p* values are listed in Supplemental Table S3.

hMSC‐TERT and primary hMSC were also compared in terms of their expression of adipocytic markers and chondrogenic markers. Of the 25 adipocyte markers that were compared (Supplemental Table S3), 12 (48%) were expressed in both hMSC‐TERT and primary hMSC and only 2 (8%) were significantly differentially expressed between the two cell types (>2 FC or <−2 FC, *p* < 0.05). Twenty‐four chrondrocyte markers were compared between hMSC‐TERT and primary hMSC, 12 (50%) were expressed in both cell types, and 3 (12.5%) were significantly differentially expressed between cell types (>2 FC or <−2 FC, *p *< 0.05) (Supplemental Table S3). These data represent baseline measurements and may thus reflect differences in cellular heterogeneity between hMSC‐TERT and primary hMSCs.

To understand the possible mechanisms associated with this, we compared gene expression of all human transcription factors (TFs) in hMSC‐TERT and primary hMSC. These were from a curated list of 1412 human TFs.^30^ We found that 722 TFs were significantly expressed in both primary hMSC and hMSC‐TERT (Illumina detection *p *< 0.01) and that 587 of these 722 (81%) were not significantly different in gene expression between the two cell populations (Fig. 2 *D*). The remaining 135 (19%) TFs were significantly differentially regulated in hMSC‐TERT relative to primary hMSC (>2 FC or <−2 FC, *p *< 0.05, Supplemental Table S4). The set of 722 TFs expressed in both primary hMSC and hMSC‐TERT is listed in Supplemental Table S5, including associated fold changes and *p* values. Biological processes that were significantly enriched in this set of 135 differentially regulated TFs included somatic stem cell population maintenance (*p *< 0.001) and the development‐associated processes embryonic digestive tract morphogenesis (*p* < 0.02) and skeletal muscle cell differentiation (*p *< 0.04). Given that we were analyzing a set of transcription factors, the most highly enriched biological processes were those relating to transcriptional regulation (*p *< 1.72 × 10^−50^).

### Telomerization enhances canonical functions of hMSC

Because telomerization has been associated with enhanced OB differentiation and bone formation, we investigated the possible molecular mechanisms associated with this. We employed the Metacore pathway analysis tool to assess biological processes and pathways regulated differently because of telomerization, and we included all differentially expressed genes through use of four fold‐change thresholds. Pathways that ranked highest in all four gene lists included the cell cycle, namely: “The metaphase checkpoint” (sum of ranks = 9); “Role of APC in cell cycle regulation” (sum of ranks = 22); “Spindle assembly and chromosome separation” (sum of ranks = 41); “Initiation of mitosis” (sum of ranks = 42); and “Role of Nek in cell cycle regulation” (sum of ranks = 95). An increase in cell cycle pathways aligns with our functional studies showing that hMSC‐TERT cells proliferate faster than primary hMSC.^9^ Other high‐ranking pathways common to all four gene lists were “SCAP/SREBP Transcriptional control of cholesterol biosynthesis” (sum of ranks = 10) and “Cytoskeleton remodeling keratin filaments” (sum of ranks = 19), both of which are also consistent with faster cell proliferation.

hMSC are known to play key roles in enhancing repair of damaged tissues, mediated through secretion of a large number of growth factors and cytokines^31^ that modulate the immune response.^31^ Comparing growth factors and immune modulatory gene profiles commonly showed similar patterns between the two cell types. However, we also observed some differences. Some growth factors showed significant change (>2 FC or <−2 FC, *p* < 0.05) in gene expression in hMSC‐TERT compared with primary hMSC cells (Table 3). Epidermal growth factor, platelet‐derived growth factor A, fibroblast growth factor 7, and transforming growth factor were downregulated, whereas fibroblast growth factor 5 was upregulated. By contrast, vascular endothelial growth factor, hepatocyte growth factor, insulin‐like growth factor, angiopoietin 1, erythropoetin, glial cell derived neurotrophic factor, C‐X‐C motif chemokine ligand 12, and interleukin 18 did not show significant differences between the cell types. With respect to immunomodulatory factors, we identified 11 genes that exhibited significant expression difference between hMSC‐TERT and primary hMSC cells (>2 FC or <−2 FC, *p* < 0.05) (Table 3). Indoleamine was downregulated as was TNFα‐stimulated gene/protein 6. Semaphorin A was upregulated, as was interleukin 10, interleukin 12A, and interleukin 1 receptor type 1. Other genes, critical for hMSC‐mediated immunosuppression, did not show significant differences in expression between the two cell types. These included chemokine ligand 2, B7‐H4, human leukocyte antigen G, leukemia inhibitory factor, galectins, heme oxygenase‐1, interleukin 6 prostaglandin E2, programmed cell death 1 ligand 1 /2, and Fas ligand.

**Table 3:** Immune Molecules Significantly Differentially Regulated Between hMSC‐TERT and Primary hMSC

Gene name	Gene symbol	Fold change	Adjusted *p* value
Growth factors			
Fibroblast growth factor 5	FGF	8.05	4.5 × 10^−5^
Transforming growth factor	TGFB2	−2.32	1.50E‐02
platelet‐derived growth factor A	PDGFA	−2.63	1.30E‐02
Epidermal growth factor	EGF	−3.98	1.00E‐03
Fibroblast growth factor 7	KGF	−6.64	6.00E‐03
Immunomodulatory factors			
Semaphorin A	SEMA3A	4.28	1.48 × 10^−6^
Interleukin 12A	IL12A	3.51	1.00E‐02
Interleukin 1 receptor type 1	IL1R1	2.91	4.80E‐02
Interleukin 10	IL10	2.15	3.2 × 10^−5^
Indoleamine	IDO	−3.96	2.00E‐02
TNFα‐stimulated gene/protein 6	TSG	−4.01	5.00E‐03

### Telomerization is associated with upregulation of cancer‐associated antigens

As noted above, there was high correlation of overall gene expression between hMSC‐TERT and primary hMSC cells. However, there were also some significant differences in gene expression; the most up‐ and downregulated genes in hMSC‐TERT relative to primary hMSC are described in Table 4. The top 10 most upregulated genes were TERT, MAGEC2, PAGE5, COL4A5, PAGE2, FAM133A, TM4SF1, CSAG1, PAGE2B, and FOLR3. The top 10 most downregulated genes were BEX1, SMOC1, TF, KCNMB1, TSPAN18, NDN, DPYSL4, SOX11, BEND5, and C20orf186.

**Table 4:** Highest Significantly Up and Downregulated Genes in hMSC‐TERT Relative to Primary hMSC

Gene symbol	Gene name	Fold change	Adjusted *p* value
TERT	Telomerase reverse transcriptase	844.10	2.84E‐11
MAGEC2	MAGE family member C2	831.43	1.59E‐09
PAGE5	PAGE family member 5	535.43	4.06E‐07
COL4A5	Collagen type IV alpha 5	317.56	4.78E‐06
PAGE2	PAGE family member 2	227.47	1.78E‐04
FAM133A	Family with sequence similarity 133 member A	215.53	1.53E‐07
TM4SF4	Transmembrane 4 L six family member 4	203.13	2.86E‐04
CSAG1	Chondrosarcoma associated gene 1	146.09	9.37E‐15
PAGE2B	PAGE family member 2B	114.60	1.11E‐06
FOLR3	Folate receptor 3 (gamma)	92.75	2.39E‐04
C20orf186	BPI fold containing family B member 4	−104.96	2.49E‐02
BEND5	BEN domain containing 5	−118.17	1.19E‐06
SOX11	SRY‐box 11	−130.27	2.84E‐06
DPYSL4	Dihydropyrimidinase‐like 4	−138.30	4.16E‐15
NDN	Necdin	−177.87	1.27E‐16
TSPAN18	Tetraspanin 18	−212.73	1.55E‐12
KCNMB1	Potassium calcium‐activated channel subfamily M regulatory beta subunit 1	−243.54	3.91E‐08
TF	Transferrin	−251.01	3.00E‐04
SMOC1	SPARC related modular calcium binding 1	−280.03	4.76E‐04
BEX1	Brain expressed X‐linked 1	−1404.44	4.03E‐07

Interestingly, 4 of the top 10 most upregulated genes in hMSC‐TERT, compared with primary hMSC, were MAGE or PAGE cancer‐associated antigens.^32^ Specifically, these were MAGEC2, PAGE5, PAGE2, and PAGE2B (Supplemental Table S6). All these genes show negligible expression levels in primary hMSC but high levels of expression in hMSC‐TERT cells, leading to up to 1800‐fold expression changes (Supplemental Fig. S1). Our group has previously reported the expression of GAGE and MAGE cancer antigens in tumorigenic telomerized hMSC‐TERT20 cells.^33^ However, the hMSC‐TERT employed in the current study are not tumorigenic, suggesting that telomerization per se may be associated with upregulation of this gene set, forming a possible “telomerization signature.”

## Discussion

In this study, we compared telomerized hMSC with primary hMSC employing a set of cell surface molecules, transcription factors and genes associated with intracellular signalling and demonstrated that telomerization preserved the molecular phenotype and maintained biological characteristics of hMSC.

Both hMSC‐TERT cells and primary hMSC shared CD markers described as the minimal criteria for defining multipotent stromal (mesenchymal) cells.^34^ These results are similar to a number of previous studies. hMSC isolated using an anti‐stro‐1 antibody, which is known to enrich for multipotent hMSC,^35^ were compared to hMSC‐TERT and reported that among 35 CD markers examined, 31 showed no significant quantitative change in expression. Similarly, Skarn and colleagues^14^ showed that the hMSC line (iMSC#3) showed no difference in cell surface markers compared with the primary hMSC.

In addition to similarities in CD marker expression, both cell types were capable of forming heterotopic bone and bone marrow organ in vivo. This has been described as the most important “stemness” criteria for hMSC. Thus, at a functional level, telomerization maintained “stemness” characteristics. Our observation corroborates results reported by Dai and colleagues,^10^ who also showed that hMSC transfected with hTERT‐CTLA4Ig formed bone in vivo.

We observed similar differentiation capacity in vitro between telomerized hMSC and primary hMSC. Whereas Dale and colleagues^36^ showed that hTERT transduction of HMSCs affected differentiation potential of the cells to varying degrees, several published studies reported that telomerization in a number of stromal cell populations maintain biological characteristics of the cells. Balducci and colleagues^37^ generated an adipose‐derived stromal cell line using transfection with hTERT and sv40 or HPV E6/E7 and the cells retained osteogenic and adipogenic differentiation potential. Wolbank and colleagues^38^ established and characterized human adipose and amnion‐derived MSC lines, using ectopic expression of hTERT, and again the cells had in addition to unaltered constellation of CD markers, similar or enhanced differentiation potential during long‐term culture (up to 87 population doublings). Also, an immortalized MSC cell line (iPMSC) derived from fetal porcine pancreas^39^ maintained their biological characteristics. We summarize these data in Table 1.

We also observed a high degree of consistency in the expression of OB, chrondrocyte, and adipocyte marker genes between the primary hMSC and hMSC‐TERT cells. A small proportion of genes relevant to the biological functions of hMSC showed significant upregulation. For example, COL4A5 was upregulated by 317.45 FC in hMSC‐TERT relative to primary hMSC. Type IV collagens are structural proteins making up the foundation for all basement membranes. These collagens form heterotrimers and interact with laminins, proteoglycans, and entactin to form an extracellular protein network.^40^ Increased levels of COL4A5 in hMSC‐TERT may thus be related to improved in vivo bone formation function of the cells.

In addition to the extensive similarities in molecular phenotype between hMSC‐TERT and primary hMSC, we observed some differences in transcription factor and cell surface marker gene expression. Most of the differences were associated with increased cell proliferation in hMSC‐TERT, suggesting that the observed enhanced biological functions in hMSC‐TERT can be explained mainly by the hTERT‐mediated anti‐replicative senescence mechanisms. The most dramatically downregulated cell surface markers upon telomerization were ICAM2 and HLA‐DRA. Both ICAM2 and HLA‐DRA are involved in an antitumor immune response.^41^, ^42^ HLA‐DRA is a MHC class II protein and presents antigens to CD4+ T cells as part of the cellular immune response. This interaction results in T‐cell activation and a strong immune response.^43^ ICAM2 is a glycoprotein that mediates adhesive interactions with T cells and has also been shown to mediate antitumor immune response in human pancreatic carcinogenesis.^42^ These effects may be caused by telomerization‐associated downregulation of antitumor response genes, which is relevant to the role of telomerization in cancer biology.^44^

A total of 19% of transcription factors analyzed showed significant change between hMSC‐TERT and primary hMSC. Two transcription factors that showed dramatic change were SOX11 (−130 FC) and TFAP2A (29 FC). SOX11 is a transcriptional regulator involved in embryonic neurogenesis and tissue modeling.^45^ De novo mutations in SOX11 have been shown to cause Coffin‐Siris, a syndrome that includes skeletal dysmorphism in humans,^46^ demonstrating its importance for skeletogenesis. Recently, Gadi and colleagues^47^ showed that SOX11C‐deficient mouse osteoblastic cell line (MC3T3‐E1) exhibit reduced cell proliferation and a significant delay in OB differentiation. TFAP2 is a member of the AP‐2 family of transcription factors, which play important roles in apoptosis, migration, and differentiation.^48^ Mutations in TFAP2A lead to branchio‐oculo‐facial syndrome, which is a cleft palate‐craniofacial disorder.^49^ TFAP2 knockout mice have severe skeletal defects and abnormalities of face and limbs, and studies demonstrate the main function of TFAP2 is suppression of terminal differentiation during embryonic development.^50^, ^51^ This implicates TFAP2 as playing a role in skeletal biology.

We have also observed upregulation of numerous cancer/testis antigens in the PAGE and MAGE gene family in hMSC‐TERT relative to primary hMSC. Increase in cancer antigen families in telomerized hMSC have been reported, including previous work from our group, which studied the tumorigenic hMSC‐TERT20.^33^, ^52^ In another study of telomerized hMSC,^14^ similar changes were observed. These cancer antigen gene functions are poorly understood, although they are frequently expressed in tumor or embryonic tissues but not in somatic tissues (as reviewed in Simpson and colleagues^53^). Knockdown of PAGE5 in melanoma cells resulted in a decrease in cell survival when cells were exposed to apoptosis‐inducing cisplatin. PAGE5 has also been shown to negatively regulate the expression of apoptotic genes and promote the survival of melanoma cells via suppression of apoptosis.^54^ The MAGE antigen MAGE2C, which was upregulated in hMSC‐TERT relative to primary hMSC, promotes both cell proliferation and viability of mast cells.^55^ Recently, CRISPR/Cas9 MAGEC2 knockout melanoma cells have been shown to have increased TNFα‐induced apoptosis.^56^ Collectively, MAGE genes have been shown to repress p53 transactivation and apoptosis and regulate cell cycle progression (as reviewed in Sang and colleagues^57^). Telomerization increases the proliferative life span of the hMSC‐TERT cells, and this may be enhanced by cancer antigen‐mediated suppression of apoptosis and regulation of cell cycle progression. These cancer antigen genes may represent a common gene expression signature induced by telomerization.

In summary, we have shown that the telomerized hMSC and primary hMSC are highly similar in both their molecular and cellular phenotypes, suggesting their suitability as a model for primary hMSC. In addition, the observed enhancement of hMSC biological function after telomerization may be mediated by increased levels of cell proliferation.

## Disclosures

All authors state that they have no conflict of interests.

## Supporting information

Supporting Figure S1.

Supporting Tables S1.

## References

[1] Abdallah BM , Bay‐Jensen AC , Srinivasan B , et al. Estrogen inhibits Dlk1/FA1 production: a potential mechanism for estrogen effects on bone turnover. J Bone Miner Res. 2011; 26(10):2548–51. 21681814 10.1002/jbmr.444 PMC3778652

[2] Sacchetti B , Funari A , Michienzi S , et al. Self‐renewing osteoprogenitors in bone marrow sinusoids can organize a hematopoietic microenvironment. Cell. 2007; 131(2):324–36. 17956733 10.1016/j.cell.2007.08.025

[3] Caplan AI , Dennis JE. Mesenchymal stem cells as trophic mediators. J Cell Biochem. 2006; 98(5):1076–84. 16619257 10.1002/jcb.20886

[4] Zaher W , Harkness L , Jafari A , Kassem M. An update of human mesenchymal stem cell biology and their clinical uses. Arch Toxicol. 2014; 88(5):1069–82. 24691703 10.1007/s00204-014-1232-8

[5] Stenderup K , Justesen J , Clausen C , Kassem M. Aging is associated with decreased maximal life span and accelerated senescence of bone marrow stromal cells. Bone. 2003; 33(6):919–26. 14678851 10.1016/j.bone.2003.07.005

[6] Stenderup K , Rosada C , Justesen J , Al‐Soubky T , Dagnaes‐Hansen F , Kassem M. Aged human bone marrow stromal cells maintaining bone forming capacity in vivo evaluated using an improved method of visualization. Biogerontology. 2004; 5(2):107–18. 15105585 10.1023/B:BGEN.0000025074.88476.e2

[7] Blasco MA. Telomere length, stem cells and aging. Nat Chem Biol. 2007; 3(10):640–9. 17876321 10.1038/nchembio.2007.38

[8] Kassem M , Abdallah BM , Yu Z , Ditzel N , Burns JS. The use of hTERT‐immortalized cells in tissue engineering. Cytotechnology. 2004; 45(1–2):39–46. 19003242 10.1007/s10616-004-5124-2 PMC3449958

[9] Simonsen JL , Rosada C , Serakinci N , et al. Telomerase expression extends the proliferative life‐span and maintains the osteogenic potential of human bone marrow stromal cells. Nat Biotechnol. 2002; 20(6):592–6. 12042863 10.1038/nbt0602-592

[10] Dai F , Yang S , Zhang F , et al. hTERT‐ and hCTLA4Ig‐expressing human bone marrow‐derived mesenchymal stem cells: in vitro and in vivo characterization and osteogenic differentiation. J Tissue Eng Regen Med. 2017; 11(2):400–11. 25047146 10.1002/term.1924

[11] Huang G , Zheng Q , Sun J , et al. Stabilization of cellular properties and differentiation mutilpotential of human mesenchymal stem cells transduced with hTERT gene in a long‐term culture. J Cell Biochem. 2008; 103(4):1256–69. 18027880 10.1002/jcb.21502

[12] Liu TM , Ng WM , Tan HS , et al. Molecular basis of immortalization of human mesenchymal stem cells by combination of p53 knockdown and human telomerase reverse transcriptase overexpression. Stem Cells Dev. 2013; 22(2):268–78. 22765508 10.1089/scd.2012.0222 PMC3545350

[13] Piper SL , Wang M , Yamamoto A , et al. Inducible immortality in hTERT‐human mesenchymal stem cells. J Orthop Res. 2012; 30(12):1879–85. 22674533 10.1002/jor.22162

[14] Skarn M , Noordhuis P , Wang MY , et al. Generation and characterization of an immortalized human mesenchymal stromal cell line. Stem Cells Dev. 2014; 23(19):2377–89. 24857590 10.1089/scd.2013.0599 PMC4172386

[15] Tsai CC , Chen CL , Liu HC , et al. Overexpression of hTERT increases stem‐like properties and decreases spontaneous differentiation in human mesenchymal stem cell lines. J Biomed Sci. 2010; 17:64. 20670406 10.1186/1423-0127-17-64 PMC2923118

[16] Kristensen LP , Chen L , Nielsen MO , et al. Temporal profiling and pulsed SILAC labeling identify novel secreted proteins during ex vivo osteoblast differentiation of human stromal stem cells. Mol Cell Proteomics. 2012; 11(10):989–1007. 22801418 10.1074/mcp.M111.012138 PMC3494153

[17] Kratchmarova I , Blagoev B , Haack‐Sorensen M , Kassem M , Mann M. Mechanism of divergent growth factor effects in mesenchymal stem cell differentiation. Science. 2005; 308(5727):1472–7. 15933201 10.1126/science.1107627

[18] Larsen KH , Frederiksen CM , Burns JS , Abdallah BM , Kassem M. Identifying a molecular phenotype for bone marrow stromal cells with in vivo bone‐forming capacity. J Bone Miner Res. 2010; 25(4):796–808. 19821776 10.1359/jbmr.091018

[19] Golub TR , Slonim DK , Tamayo P , et al. Molecular classification of cancer: class discovery and class prediction by gene expression monitoring. Science. 1999; 286(5439):531–7. 10521349 10.1126/science.286.5439.531

[20] Twine NA , Chen L , Pang CN , Wilkins MR , Kassem M. Identification of differentiation‐stage specific markers that define the ex vivo osteoblastic phenotype. Bone. 2014; 67c:23–32. 10.1016/j.bone.2014.06.027 24984278

[21] Harkness L , Zaher W , Ditzel N , Isa A , Kassem M. CD146/MCAM defines functionality of human bone marrow stromal stem cell populations. Stem Cell Res Ther. 2016; 7:4. 26753846 10.1186/s13287-015-0266-z PMC4710006

[22] Kassem M , Mosekilde L , Eriksen EF. 1,25‐dihydroxyvitamin D3 potentiates fluoride‐stimulated collagen type I production in cultures of human bone marrow stromal osteoblast‐like cells. J Bone Miner Res. 1993; 8(12):1453–8. 8304046 10.1002/jbmr.5650081207

[23] Harkness L , Mahmood A , Ditzel N , Abdallah BM , Nygaard JV , Kassem M. Selective isolation and differentiation of a stromal population of human embryonic stem cells with osteogenic potential. Bone. 2011; 48(2):231–41. 20869473 10.1016/j.bone.2010.09.023

[24] Abdallah BM , Ditzel N , Kassem M. Assessment of bone formation capacity using in vivo transplantation assays: procedure and tissue analysis. Methods Mol Biol. 2008; 455:89–100. 18463812 10.1007/978-1-59745-104-8_6

[25] R Core Team. R: a language and environment for statistical computing. Vienna, Austria: R Foundation for Statistical Computing; 2013.

[26] Ritchie ME , Phipson B , Wu D , et al. limma powers differential expression analyses for RNA‐sequencing and microarray studies. Nucleic Acids Res. 2015; 43(7):e47. 25605792 10.1093/nar/gkv007 PMC4402510

[27] Huang da W , Sherman BT , Lempicki RA . Systematic and integrative analysis of large gene lists using DAVID bioinformatics resources. Nat Protoc. 2009; 4(1):44–57. 19131956 10.1038/nprot.2008.211

[28] Ziegler‐Heitbrock HW , Ulevitch RJ. CD14: cell surface receptor and differentiation marker. Immunol Today. 1993; 14(3):121–5. 7682078 10.1016/0167-5699(93)90212-4

[29] Al‐Nbaheen M , Vishnubalaji R , Ali D , et al. Human stromal (mesenchymal) stem cells from bone marrow, adipose tissue and skin exhibit differences in molecular phenotype and differentiation potential. Stem Cell Rev. 2013; 9(1):32–43. 22529014 10.1007/s12015-012-9365-8 PMC3563956

[30] Vaquerizas JM , Kummerfeld SK , Teichmann SA , Luscombe NM. A census of human transcription factors: function, expression and evolution. Nat Rev Genet. 2009; 10(4):252–63. 19274049 10.1038/nrg2538

[31] Ma S , Xie N , Li W , Yuan B , Shi Y , Wang Y. Immunobiology of mesenchymal stem cells. Cell Death Differ. 2014; 21(2):216–25. 24185619 10.1038/cdd.2013.158 PMC3890955

[32] Fratta E , Coral S , Covre A , et al. The biology of cancer testis antigens: putative function, regulation and therapeutic potential. Mol Oncol. 2011; 5(2):164–82. 21376678 10.1016/j.molonc.2011.02.001 PMC5528287

[33] Gjerstorff M , Burns JS , Nielsen O , Kassem M , Ditzel H. Epigenetic modulation of cancer‐germline antigen gene expression in tumorigenic human mesenchymal stem cells: implications for cancer therapy. Am J Pathol. 2009; 175(1):314–23. 19498007 10.2353/ajpath.2009.080893 PMC2708817

[34] Dominici M , Le Blanc K , Mueller I , et al. Minimal criteria for defining multipotent mesenchymal stromal cells. The International Society for Cellular Therapy position statement. Cytotherapy. 2006; 8(4):315–7. 16923606 10.1080/14653240600855905

[35] Gothard D , Greenhough J , Ralph E , Oreffo RO. Prospective isolation of human bone marrow stromal cell subsets: a comparative study between Stro‐1‐, CD146‐ and CD105‐enriched populations. J Tissue Eng. 2014; 5:2041731414551763. 25383172 10.1177/2041731414551763 PMC4221949

[36] Dale TP , de Castro A , Kuiper NJ , Parkinson EK , Forsyth NR. Immortalisation with hTERT impacts on sulphated glycosaminoglycan secretion and immunophenotype in a variable and cell specific manner. PloS One. 2015; 10(7):e0133745. 26196672 10.1371/journal.pone.0133745 PMC4510558

[37] Balducci L , Blasi A , Saldarelli M , et al. Immortalization of human adipose‐derived stromal cells: production of cell lines with high growth rate, mesenchymal marker expression and capability to secrete high levels of angiogenic factors. Stem Cell Res Ther. 2014; 5(3):63. 24887516 10.1186/scrt452 PMC4055112

[38] Wolbank S , Stadler G , Peterbauer A , et al. Telomerase immortalized human amnion‐ and adipose‐derived mesenchymal stem cells: maintenance of differentiation and immunomodulatory characteristics. Tissue Eng Part A. 2009; 15(7):1843–54. 19125642 10.1089/ten.tea.2008.0205 PMC3092731

[39] Cao H , Chu Y , Zhu H , et al. Characterization of immortalized mesenchymal stem cells derived from foetal porcine pancreas. Cell Prolif. 2011; 44(1):19–32. 21199007 10.1111/j.1365-2184.2010.00714.x PMC6496693

[40] Timpl R , Brown JC. Supramolecular assembly of basement membranes. Bioessays. 1996; 18(2):123–32. 8851045 10.1002/bies.950180208

[41] Vesely MD , Kershaw MH , Schreiber RD , Smyth MJ. Natural innate and adaptive immunity to cancer. Annu Rev Immunol. 2011; 29:235–71. 21219185 10.1146/annurev-immunol-031210-101324

[42] Hiraoka N , Yamazaki‐Itoh R , Ino Y , et al. CXCL17 and ICAM2 are associated with a potential anti‐tumor immune response in early intraepithelial stages of human pancreatic carcinogenesis. Gastroenterology. 2011; 140(1):310–21. 20955708 10.1053/j.gastro.2010.10.009

[43] Blum JS , Wearsch PA , Cresswell P. Pathways of antigen processing. Annu Rev Immunol. 2013; 31:443–73. 23298205 10.1146/annurev-immunol-032712-095910 PMC4026165

[44] Li Y , Tergaonkar V. Noncanonical functions of telomerase: implications in telomerase‐targeted cancer therapies. Cancer Res. 2014; 74(6):1639–44. 24599132 10.1158/0008-5472.CAN-13-3568

[45] Potzner MR , Tsarovina K , Binder E , et al. Sequential requirement of Sox4 and Sox11 during development of the sympathetic nervous system. Development. 2010; 137(5):775–84. 20147379 10.1242/dev.042101 PMC2827687

[46] Tsurusaki Y , Koshimizu E , Ohashi H , et al. De novo SOX11 mutations cause Coffin‐Siris syndrome. Nat Commun. 2014; 5:4011. 24886874 10.1038/ncomms5011

[47] Gadi J , Jung SH , Lee MJ , et al. The transcription factor protein Sox11 enhances early osteoblast differentiation by facilitating proliferation and the survival of mesenchymal and osteoblast progenitors. J Biol Chem. 2013; 288(35):25400–13. 23888050 10.1074/jbc.M112.413377 PMC3757203

[48] Eckert D , Buhl S , Weber S , Jager R , Schorle H. The AP‐2 family of transcription factors. Genome Biol. 2005; 6(13):246. 16420676 10.1186/gb-2005-6-13-246 PMC1414101

[49] Milunsky JM , Maher TA , Zhao G , et al. TFAP2A mutations result in branchio‐oculo‐facial syndrome. Am J Hum Genet. 2008; 82(5):1171–7. 18423521 10.1016/j.ajhg.2008.03.005 PMC2427243

[50] Zhang J , Hagopian‐Donaldson S , Serbedzija G , et al. Neural tube, skeletal and body wall defects in mice lacking transcription factor AP‐2. Nature. 1996; 381(6579):238–41. 8622766 10.1038/381238a0

[51] Schorle H , Meier P , Buchert M , Jaenisch R , Mitchell PJ. Transcription factor AP‐2 essential for cranial closure and craniofacial development. Nature. 1996; 381(6579):235–8. 8622765 10.1038/381235a0

[52] Burns JS , Abdallah BM , Guldberg P , Rygaard J , Schroder HD , Kassem M. Tumorigenic heterogeneity in cancer stem cells evolved from long‐term cultures of telomerase‐immortalized human mesenchymal stem cells. Cancer Res. 2005; 65(8):3126–35. 15833842 10.1158/0008-5472.CAN-04-2218

[53] Simpson AJ , Caballero OL , Jungbluth A , Chen YT , Old LJ. Cancer/testis antigens, gametogenesis and cancer. Nat Rev Cancer. 2005; 5(8):615–25. 16034368 10.1038/nrc1669

[54] Nylund C , Rappu P , Pakula E , et al. Melanoma‐associated cancer‐testis antigen 16 (CT16) regulates the expression of apoptotic and antiapoptotic genes and promotes cell survival. PloS One. 2012; 7(9):e45382. 23028975 10.1371/journal.pone.0045382 PMC3448647

[55] Yang B , O'Herrin S , Wu J , et al. Select cancer testes antigens of the MAGE‐A, −B, and −C families are expressed in mast cell lines and promote cell viability in vitro and in vivo. J Invest Dermatol. 2007; 127(2):267–75. 16960553 10.1038/sj.jid.5700548

[56] Wang J , Song X , Guo C , Wang Y , Yin Y. Establishment of MAGEC2‐knockout cells and functional investigation of MAGEC2 in tumor cells. Cancer Sci. 2016; 107(12):1888–97. 27636589 10.1111/cas.13082 PMC5198962

[57] Sang M , Wang L , Ding C , et al. Melanoma‐associated antigen genes—an update. Cancer Lett. 2011; 302(2):85–90. 21093980 10.1016/j.canlet.2010.10.021

