# Supplementary figures and images for: Molecular Phenotyping of Telomerized Human Bone Marrow Skeletal Stem Cells Reveals a Genetic Program of Enhanced Proliferation and Maintenance of Differentiation Responses
Source: JBMR Plus. 2018 May 24;2(5):257–67. doi: 10.1002/jbm4.10050 (PMC6139702; doi:10.1002/jbm4.10050)

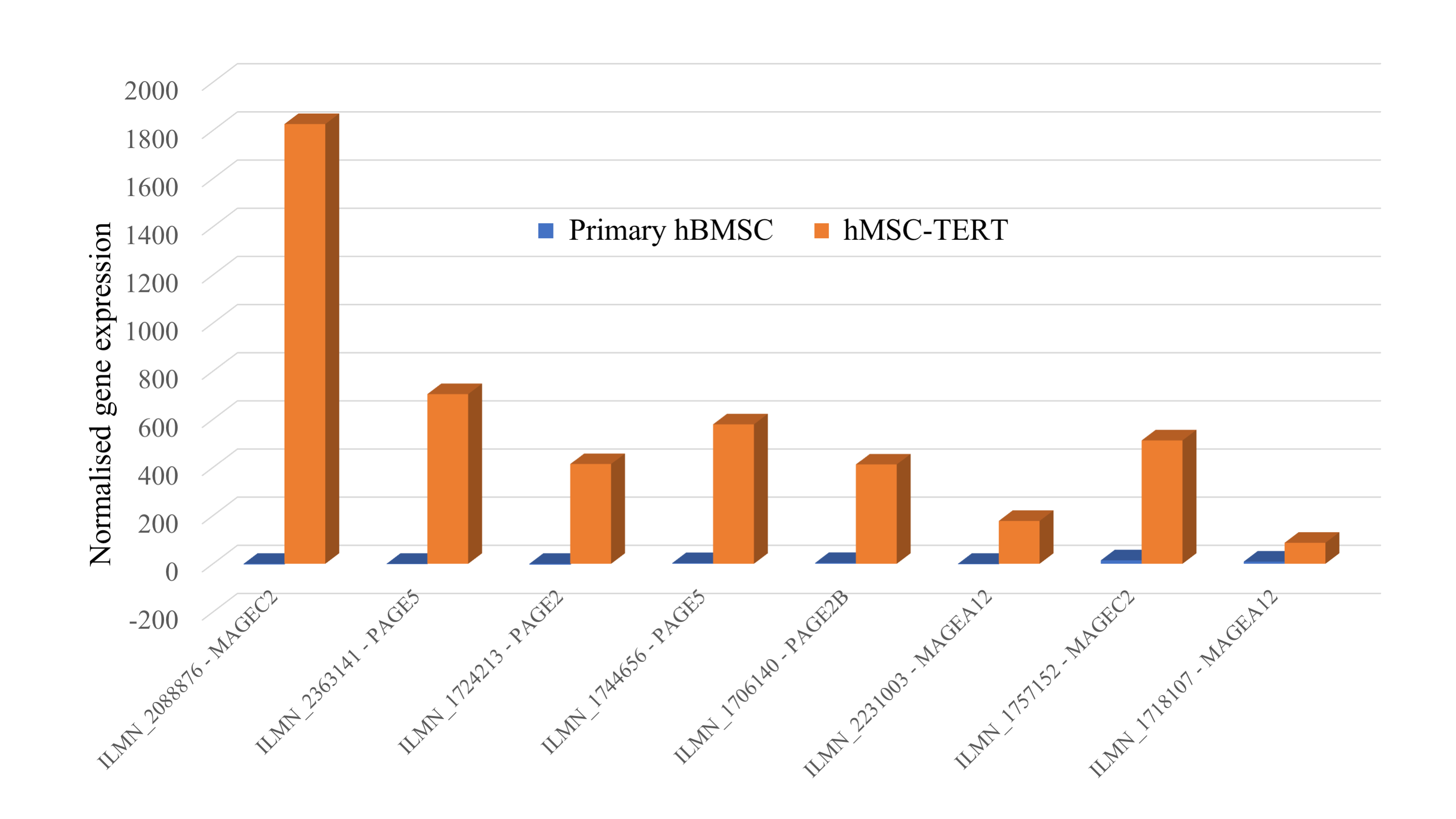

Supplement: Supplementary file 1 — Supporting Figure S1. [file JBM4-2-257-s001.tiff]
